# Optimization of Phycocyanobilin Synthesis in *E. coli* BL21: Biotechnological Insights and Challenges for Scalable Production

**DOI:** 10.3390/genes15081058

**Published:** 2024-08-12

**Authors:** Julia Esclapez, Laura Matarredona, Guillermo Zafrilla, Mónica Camacho, María-José Bonete, Basilio Zafrilla

**Affiliations:** 1 Department of Biochemistry and Molecular Biology and Soil Science and Agricultural Chemistry, Faculty of Science, University of Alicante, Carretera de San Vicente del Raspeig s/n, 03690 Alicante, Spain; julia.esclapez@ua.es (J.E.); laura.sm@ua.es (L.M.); gz@globalbiotech.es (G.Z.); camacho@ua.es (M.C.); 2 Global BioTech SL, C/Padre Manjón Nº2, 03560 Alicante, Spain

**Keywords:** phycocyanobilin, BL21 (DE3), biosynthesis, HO1, PCYA, heterologous expression, bioreactor, optimization

## Abstract

Phycocyanobilin (PCB) is a small chromophore found in certain phycobiliproteins, such as phycocyanins (PCs) and allophycocyanins (APCs). PCB, along with other phycobilins (PBs) and intermediates such as biliverdin (BV) or phycoerythrobilin (PEB), is attracting increasing biotechnological interest due to its fluorescent and medicinal properties that allow potential applications in biomedicine and the food industry. This study aims to optimize PCB synthesis in *Escherichia coli* BL21 (DE3) and scale the process to a pre-industrial level. Parameters such as optimal temperature, inducer concentration, initial OD_600_, and stirring speed were analyzed in shake flask cultures to maximize PCB production. The best results were obtained at a temperature of 28 °C, an IPTG concentration of 0.1 mM, an initial OD_600_ of 0.5, and an orbital shaking speed of 260 rpm. Furthermore, the optimized protocol was scaled up into a 2 L bioreactor batch, achieving a maximum PCB concentration of 3.8 mg/L. Analysis of the results revealed that biosynthesis of exogenous PBs in *Escherichia coli* BL21 (DE3) is highly dependent on the metabolic burden of the host. Several scenarios, such as too rapid growth, high inducer concentration, or mechanical stress, can advance entry into the stationary phase. That progressively halts pigment synthesis, leading, in some cases, to its excretion into the growth media and ultimately triggering rapid degradation by the host. These conclusions provide a promising protocol for scalable PCB production and highlight the main biotechnological challenges to increase the yields of the process.

## 1. Introduction

**Figure 1:**
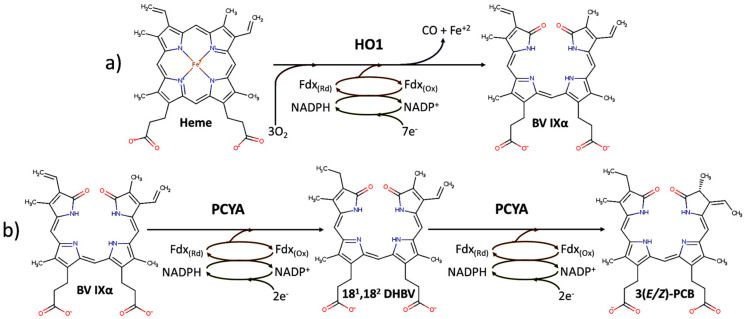
Scheme of the two enzymatic steps and the related enzymes involved in the biosynthesis of PCB (phycocyanobilin) from heme. (**a**) Production of BV IXα (biliverdin) in the first enzymatic steep by heme oxygenase (HO1). (**b**) Production of PCB in the second enzymatic steep by phycocyanobilin:ferredoxin oxidoreductase (PCYA).

Phycocyanobilin (PCB) is a bluish pigment covalently bounded to α and β subunits of some phycobiliproteins (PBPs) such as phycocyanin (PC) or allophycocyanin (APC) [1,2,3]. Structurally, PCB is an open-chain tetrapyrrole that acts as these PBPs’ primary chromophore [4]. Its spectroscopic properties enable specific taxa of photosynthetic organisms to utilize wavelengths near the red that are not useful to traditional pigments such as chlorophylls or carotenoids [5]. Regarding their biosynthesis, PCB and other phycobilins (PBs), such as phycoerithrobilin (PEB) and phycoviolobilin (PVB), are derived from an alternative pathway of heme degradation. This pathway consists of two enzymatic steps, which, in the case of PCB, are catalyzed by two different enzymes [6]. Firstly, heme groups are reduced to biliverdin IXα in the presence of O_2_ by the heme oxygenase 1 (HO1). As a result of this catalysis, CO and Fe^+2^ molecules are released (Figure 1a). This first step is common to all pathways derived from the cleavage of the heme ring, encompassing both those related to the formation of all PBs and the purely degradative pathway that proceeds with the production of bilirubin and derivatives (urobilinogen, urobilin, stercobilin, etc.). Secondly, a phycocyanobilin:ferredoxin oxidoreductase (PCYA), reduces BV to PCB, passing through 18^1^,18^2^-dihydrobiliverdin (DHBV) as an intermediary compound (Figure 1b). Both HO1 and PCYA are ferredoxin-dependent enzymes, and the electrons needed in each step are supplied by a ferredoxin-NADP^+^ reductase (FNR), so the biosynthesis is strongly dependent on the reducing power availability (NADPH) in the cell.

In recent years, PCB has progressively increased its biotechnological relevance because multiple applications in the food industry have been proposed due to its natural blue color [7]. Furthermore, numerous studies postulate that this pigment can become a powerful antioxidant and antitumor agent, which has generated significant commercial interest from the pharmaceutical and nutritional supplement industries [3]. Some studies have even proposed PCB as a potential protector against Alzheimer’s disease due to its antioxidant, anti-inflammatory, and immunomodulatory properties [8]. However, PCB production methods do not go beyond simple laboratory-scale research assays. Most of these methods try to break up the chromophore from the holo-PC through acidic hydrolysis and mainly through solvents such as methanol and ethanol, together with high temperatures [9]. However, these methodologies are usually tedious, inefficient, and expensive, using solvents that can be dangerous for health and the environment. A second option for the industrial production of this PCB involves the heterologous co-expression of HO1 and PCYA in a standardized host such as *Escherichia coli* BL21 (DE3) and coupling in vivo the enzymatic pathway using the host’s precursors. Recent works show several significant advances in this field, achieving variable production titers of PCB [10,11,12,13,14,15,16]. Nevertheless, several key factors for the heterologous synthesis remain unclear, and frequently, the obtained results are not reproducible in other laboratories. In this study, the key factors (medium composition, O_2_ availability, presence of precursors, concentration of the inducer, etc.) that determine the ability of the host to initiate, maintain, and preserve the production of this xenobiotic molecule are experimentally explored. Beyond this, we apply the main conclusions derived from 250 mL shake flask studies to test the PCB biosynthesis at the pre-industrial level using a 2 L bioreactor.

## 2. Materials and Methods

### 2.1. Strains and Genes

*E. coli* DH5α (ThermoFisher Scientific, Waltham, MA, USA) was used for molecular biology procedures, while *E. coli* Bl21 (DE3) (Novagen-Merck, Darmstadt, Germany) was used as a host for the heterologous expression of proteins and as an enzymatic engineering scaffold. The genes encoding HO1 and PCYA enzymes were amplified from the *Synechococcus elongatus* PCC7942 and *Synechocystis sp.* PCC6803 cyanobacterium genomes. Both species were obtained from the ATCC culture collection (Manassas, VA, USA). After the first set of expression assays, the expression levels using native sequences were insufficient, and no color was detected in the pellets. For this reason, the codon usage was optimized for translation in *E. coli* BL21 (DE3) using the GenSmart^TM^ online tool from GenScript Biotech Corporation (Pennington, NJ, USA) (Figure S1). The resulting ORFs, named *ho1*^1^/*pcyA*^1^ (from *S. elongatus*) and *ho1*^2^/*pcyA*^2^ (from *Synechocystis* sp.), were artificially synthesized by the company Isogen Lifesciences B.V. (Veldzigt, The Nederthlands), including appropriate restriction sites for further manipulation.

### 2.2. Vectors and Genetic Constructions

The two *ho1* genes were individually inserted in the MCS (Multiple cloning site) II of pCDFDuet^TM^ vectors purchased from Novagen-Merck (Darmstadt, Germany). In the same way, two *pcyA* genes were harbored in the MCS II of pETDuet^TM^ vectors from the same provider. Thus, all the possible combinations between the four different constructions could be assessed. Finally, a *pcyA*^2^/*ho1*^2^ tandem was harbored in the MCS I and MCS II of the pETDuet^TM^ vector. After confirming that the PCB titer was identical to that obtained with combined vectors, pETDuet–*pcyA*^2^/*ho1*^2^ was chosen to carry out the subsequent assays.

### 2.3. Growth Media, Precursors, and Culture Systems

The components of the different growth media are described in Table 1. All the reactives were purchased from AppliChem Panreac (Castellar del Vallés, Spain). Briefly, the assayed media used to improve the PCB synthesis were LB (Luria Bertani), TB (Terrific Broth), and MM9 (Modified M9 medium). MM9 medium contained 30 g/L of glycerol as the primary carbon source and 10 g/L of casein peptone as the nitrogen source. All the potential precursors were acquired from SigmaAldrich-Merk (Darmstadt, Germany). The assayed concentrations were 15 mM α-aminolevulinic acid (ALA), 10 µM Hemin (HEM), 15 mM glutamic acid (GA), 2 mM nicotine adenine dinucleotide phosphate (NADP^+^), and 1 g/L of ascorbic acid (VC). To ensure the stability of the plasmids, the strains transformed with the pETDuet^TM^ and/or pCDFDuet^TM^ vectors were selected with 60 µg/mL ampicillin and 40 µg/mL streptomycin (AppliChem Panreac, Castellar del Vallés, Spain), respectively, or by a combination of them. Two different culture systems were employed in this study. The OD_600_ of the cultures was determined using a 6131 Biophotometer (Eppendorf, Hamburg, Germany). On the one hand, the optimization of the PCB biosynthesis was conducted in 250 mL shake flasks containing 75 mL of medium. Conversely, all the batch assays in the bioreactor were carried out in a Biostat^®^ B (B. Braun Biotech, Melsungen, Germany) harboring a 2 L vessel with 1.2 L of MM9 medium.

### 2.4. Protein Expression and Biosynthesis Assays of PBs in Flasks and Batch Bioreactor

All synthesis assays started from a fresh pre-culture obtained by inoculating 75 mL of LB with a single colony from a fresh plate of transformants. As discussed later, temperatures close to 37 °C (optimal for the host’s growth) were detrimental to pigment synthesis. To avoid this issue, this inoculum was grown at 34 °C, which was the highest temperature in the subsequent optimization assays. This pre-culture was grown overnight at 180 rpm and used to inoculate the different expression assays at an OD_600_ of 0.1. After that, the cultures were grown under assayed conditions in each case until reaching the selected OD_600_ for the induction. As main determining factors for the PCB and BV synthesis, the effect of the induction OD_600_ (0.15, 0.3, 0.5, 0.8, and 1.0); the concentration of IPTG (isopropyl β-d-1-thiogalactopyranoside) as inducer (0.05, 0.1, 0.3, and 0.8 mM); the temperature of the culture (18, 28 and 34 °C) during biosynthesis, and the shaking speed (60, 180, and 250 rpm) were analyzed. When one of these four factors was examined, the other remained at their optimal points (28 °C, 0.1 mM IPTG, 250 rpm of shaking, and OD_600_ of 0.5). A control culture was kept under the same conditions for each assay, but the induction step was avoided. For the 2 L assays in a batch bioreactor, the culture was initially seeded at an OD_600_ of 0.25 and allowed to grow at 34 °C with 3 L/min of airflow and 200 rpm of stirring until reaching an OD_600_ of 1.5 units. At this point, it was induced with 0.1 mM of IPTG, and the temperature was reduced to 30 °C. After 4 h post-induction, the temperature was decreased to 28 °C to gradually slow the host’s metabolism. Due to the phosphate buffer incorporated into the MM9 medium, the pH remained constant between 6.8 and 7.2 units, so no corrections were needed throughout the assays. The pH, rpm, pO_2_, and temperature were continuously monitored during the batch. In both 250 mL flakes and 2 L batches, the total induction time was 8 h.

### 2.5. Monitoring of Pigment Synthesis

In all the assays, 2.5 mL of culture was taken every hour after induction. From this volume, 0.5 mL was employed to measure the OD_600_, and the remaining 2 mL were centrifuged. To avoid their degradation, the resulting pellets were stored at 4 °C until the visual analysis was carried out at the end of each assay. In this analysis, all collected pellets were visually compared with the control to establish a chronological synthesis line.

### 2.6. Quantification of PBs at Final Point

After 8 h of induction, the last OD_600_ value was logged, and 50 mL of the culture was centrifuged. The pellet was cooled to 4 °C, and the pigment content was extracted by adding 3 mL of methanol, vigorous shaking, and incubation at 4 °C overnight. The methanolic extract was separated from the cellular debris by centrifugation (10 min, 14,000 rpm) at 4 °C. Finally, this extract was acidified by adding 5% (v/v) 10 N HCl. The samples were filtered to 0.2 µm and then analyzed by spectrophotometry or HPLC. For quantification, the extinction coefficient of 37.9 mM^−1^ ·cm^−1^ (ε_680_) was used for PCB, and 30.8 mM^−1^ ·cm^−1^ (ε_695_) was used for BV-IXα [17]. For HPLC quantification, a Kinetex C8 column (Phenomenex, Torrance, USA) was employed under the control of an Agilent 1260 Infinity I system. The column was equilibrated with 50:50 water/acetone mobile phase and 0.06% acetic acid. During separation, 50 µL of acidified sample was isocratically eluted at 0.8 mL/min at room temperature for 18 min with the same solvent. The retention time for PCB and BV was 12.1 and 14.6 min, respectively, detected by a VWD (G1314B) detector at 600 nm. SigmaAldrich-Merck (Darmstadt, Germany) provided the PCB and BV standards to enable pigment identification and build the equipment calibration. 

## 3. Results

### 3.1. Selection of the Genes

**Figure 2:**
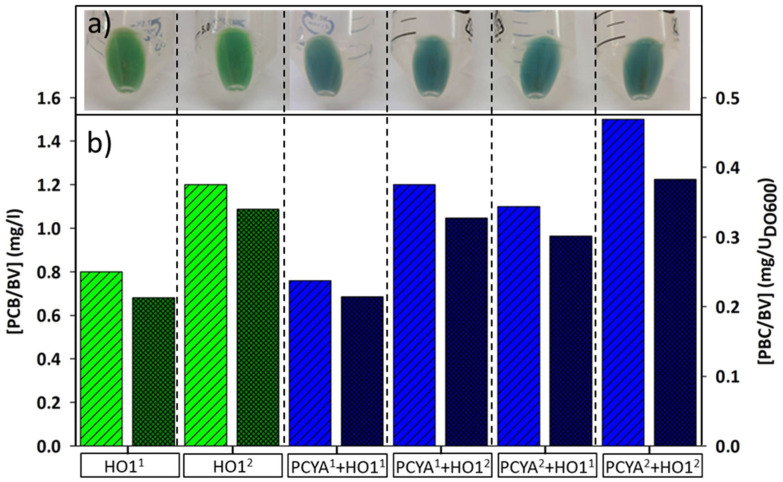
Results derived from gene combination assays. The concentration of BV (biliverdin) and PCB (phycocyanobilin) in the *Escherichia coli* BL21 (DE3) biomass is displayed as the macroscopic appearance (**a**) and numerical values (**b**). The X-axis shows the expressed gene/genes, while in the Y-axis, the concentration of BV (green bars) and PCB (blue bars) is given both per liter of culture (left bars) and per unit of OD_600_ (right bars).

The sequences of genes required for PCB synthesis were obtained from both *S. elongatus* PCC7942 and *Synechochistys sp*. PCC6830. After the codon optimization, the synthesis of both BV and PCB was carried out by using different combinations of the four genes. BV synthesis was assayed by comparing the efficiency of pCDF-*ho1^1^* versus pCDF-*ho1^2^*. On the other hand, PCB synthesis was analyzed by comparing the four possible combinations of the previous vectors with pET-*pcyA^1^* and pET-*pcyA^2^*. A standard set of synthesis conditions (30 °C, 180 rpm, and 0.1 mM IPTG in MM9 medium) was chosen for this cluster-off assay. The best titer of PCB was obtained by combining the two genes (*pcyA*^2^/*ho1*^2^) from *Synechocystis sp*. after 8 h of induction (Figure 2a,b). The enzymes derived from this cyanobacterium fit slightly better in the metabolism of *E. coli* BL21 (DE3).

### 3.2. Detection and Quantification of the Pigments

**Figure 3:**
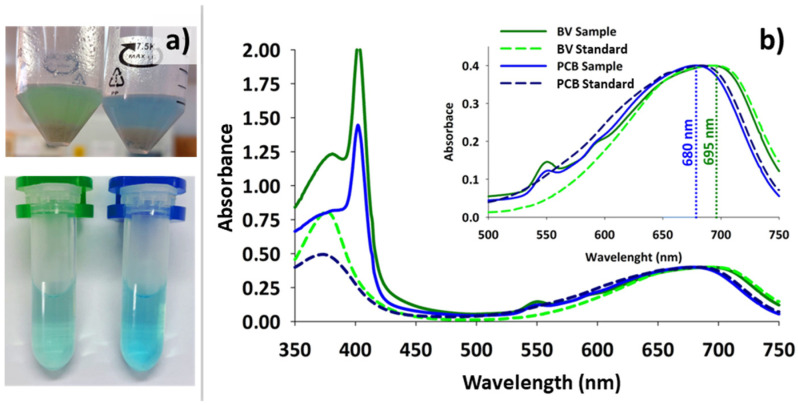
(**a**) Appearance of BV (biliverdin) and PCB (phycocyanobilin) extracted from the colored pellets under neutral (top) and acidified (bottom) conditions. (**b**) VIS spectrum of the acidified methanolic extracts from the pellets (solid lines) and the commercial standards (dashed lines) of BV (green) and PCB (blue). The shift in the two maxima is shown in the upper inset.

At the end point of all the assays, the synthesis of both BV and PCB was visually evaluated by centrifugation of 50 mL of the culture (Figure 2a). After extracting the obtained pellets, the pigment identification and routine quantification were carried out by spectrophotometry (Figure 3a,b). The synthesized BV produced an absorption peak at 695 nm in acidic methanol, while PCB displayed a maximum at 680 nm. Finally, both compounds were successfully identified and quantified by HPLC (Figure S2).

### 3.3. Effect of the Growth Medium and Presence of Precursors in the Biosynthesis

**Figure 4:**
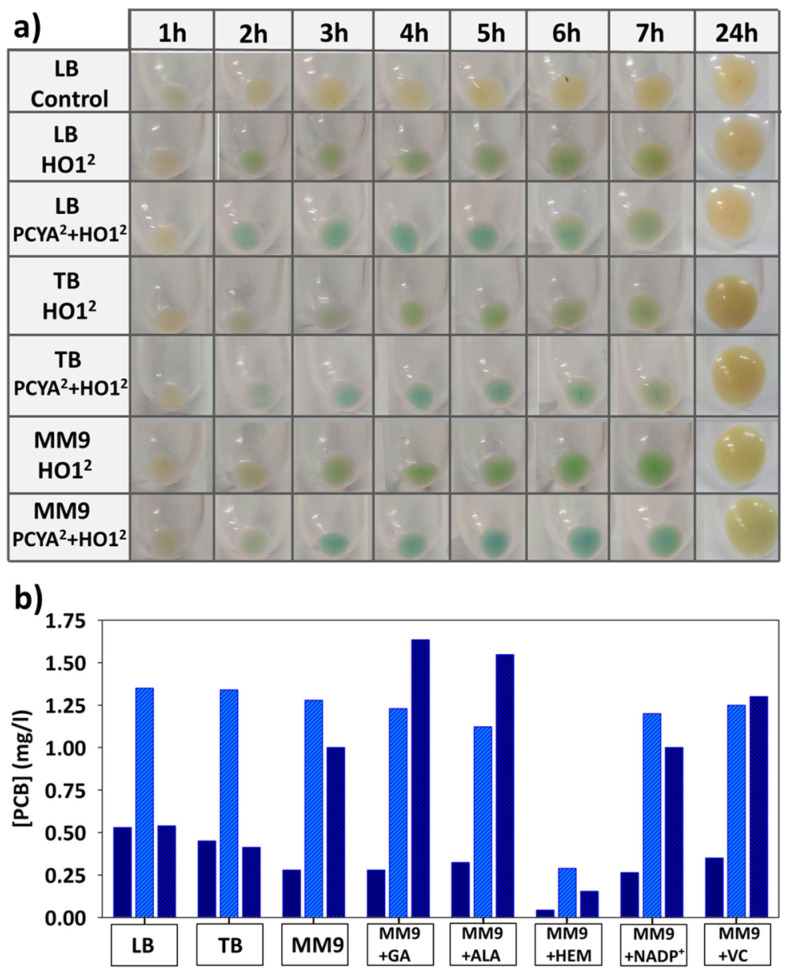
Effect of the growth medium and precursors on BV (biliverdin) and PCB (phycocyanobilin) biosynthesis. (**a**) Time-course evolution of BV and PCB at the visual level in the three tested media. (**b**) Quantitative effect of the three tested media and the presence of precursors on PCB biosynthesis. The left, center, and right columns show PCB concentration in *Escherichia coli* BL21 (DE3) cells at 3, 5, and 8 h, respectively, from the moment of induction.

The effectiveness of LB, TB, and MM9 media in providing the required precursors to synthesize BV and PCB was evaluated. Although they all generated similar levels of pigments in the first hours of induction, the assays developed in LB and TB suffered a drastic degradation of the fresh pigments after the fifth hour from the induction point (Figure 4a). As discussed in later paragraphs, this rapid breakdown of the exogenous metabolite after the first hours of the process was observed under several conditions. On the contrary, the MM9 medium seemed to confer certain stability to the pigments so that, even after 24 h from induction, a slight blue or green tone could still be detected. This effect of the MM9 medium was enhanced with the addition of GA, proposed as a possible precursor of heme group synthesis in *E. coli* [18]. Under these conditions, PCB was continuously synthesized for over 8 h, reaching the best results achieved in flasks with 1.7 mg/L. Furthermore, supplementing LB and TB media with GA did not yield any noticeable enhancement or protective effect, possibly due to the abundance of other nitrogen sources in these media. On the other hand, the addition of the other assessed precursors or intermediates, such as ALA, NADP^+^, HEM, or VC, had no significant effect on or improvement in the production rate or stability of the exogenous pigments (Figure 4b). Furthermore, the direct addition of heme as hemin led to potent inhibition of the enzymatic pathway, causing the synthesis values to drop to almost zero; in these cases, the pellet became dark brown.

### 3.4. Optimization of Parameters for the PCB Biosynthesis

Once the culture medium was optimized, the conditions for PCB synthesis during induction were adjusted for (i) temperature, (ii) IPTG concentration, (iii) initial OD_600_, and (iv) agitation.

#### 3.4.1. Temperature

**Figure 5:**
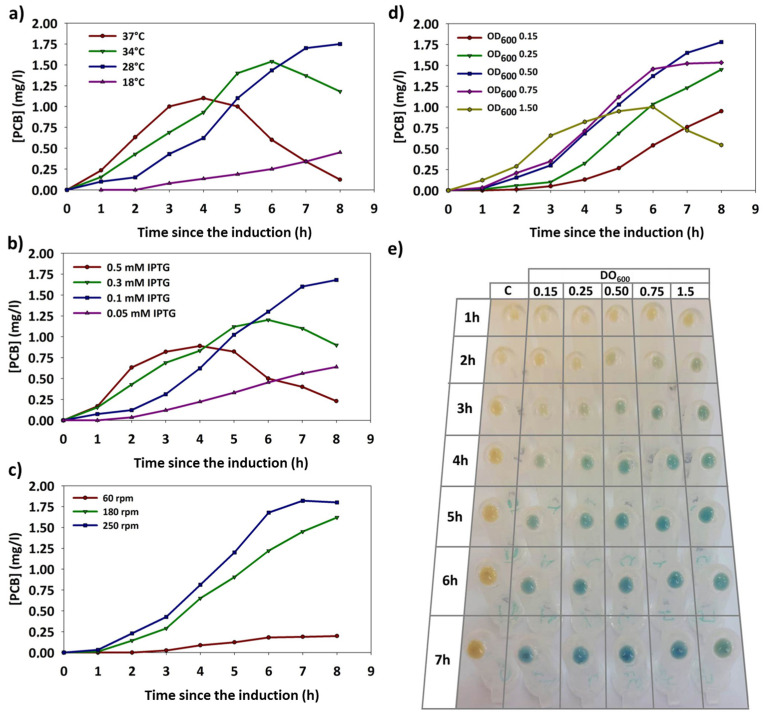
Effect of (**a**) temperature, (**b**) IPTG concentration, (**c**) shaking rate, and (**d**) induction OD_600_ on PCB (phycocyanobilin) biosynthesis. (**e**) Visual synopsis of the effect of the different values of OD_600_ at induction on PCB biosynthesis over time.

Temperature is a crucial factor influencing the metabolic rate of microorganisms. The results indicated that at optimal growth temperatures (34–37 °C), *E. coli* BL21 (DE3) developed a very high metabolic rate. This led to an explosive synthesis of recombinant enzymes and rapid pathway coupling for pigment production. Under these conditions, a visible blue color appeared in the pellets just one hour after induction. However, this high metabolic rate posed challenges for maintaining sustained pigment production. PCB concentration peaked between 4 and 5 h post-induction, followed by abrupt degradation (Figure 5a). This degradation is likely due to a rapid transition of the culture toward the stationary phase. In this scenario, *E. coli* BL21 (DE3) must alter its metabolism to face a stressful panorama. Precursors for pigment synthesis (e.g., heme groups, O_2_) are rapidly depleted or oxidized (NADP^+^) at this temperature. This retargeting of the metabolism halts new pigment synthesis and activates degradative mechanisms to recycle freshly synthesized pigments. Additionally, the explosive synthesis of recombinant proteins, even more at high IPTG concentrations, resulted in large amounts of inclusion bodies containing significant fractions of both enzymes (Figure S3), creating another metabolic burden that conducted the culture to an early stationary phase. In contrast, cultures grown at 18 °C exhibited a prolonged latency (>3 h) from induction to visible pigment detection in the pellet. The reduced metabolic activity at this temperature delayed the expression of T7 RNA polymerase and, subsequently, the production of recombinant enzymes. The activities of HO1 and PCYA were also reduced, making it challenging to achieve high PCB concentrations. However, the few synthesized PCBs showed minimal degradation, likely due to the slow transition toward the stationary phase and the concurrent reduction of degradative pathways. In summary, the optimal temperature for PCB synthesis in the flask was 28 °C, where the pigment was synthesized and accumulated consistently for up to 8 h, reaching the highest detected concentrations. It is important to note that temperature and IPTG concentrations are closely interrelated, exhibiting a synergistic relationship.

#### 3.4.2. IPTG Concentration

The IPTG concentration is also crucial for achieving sustained pigment synthesis. Parallel to temperature, IPTG concentrations at 0.3 mM or higher resulted in rapid expression of the recombinant proteins, allowing pigment detection within the first hours of the assay. This feature was more evident with increasing temperature. Moreover, these high IPTG concentrations seemed to interfere with the normal metabolism of the host, increasing the metabolic burden and becoming a source of stress, which originates an early entry into the stationary growth phase [19]. Combined with temperatures of 34–37 °C, these high concentrations of IPTG resulted in more than 50% of the recombinant enzymes expressed as inclusion bodies (Figure S3). Consequently, this situation resulted in an abrupt stop and rapid degrading of the freshly synthesized pigments a few hours after the induction point.

Conversely, IPTG concentrations below 0.1 mM avoided its toxic effect. Still, they also resulted in significantly delayed expression of recombinant enzymes, and the pigment synthesis was negligible until several hours after induction (Figure 5b). Under these conditions, the culture grew rapidly and reached the stationary phase with low pigment accumulation. The degradative process began at this stage, and the small amounts of synthesized pigment were finally degraded. The best results were obtained with 0.1 mM of IPTG. This concentration was enough to trigger adequate recombinant enzyme expression but significantly avoided the toxic effect of the inducer. The synthesis could be sustained for over 8 h in these conditions, provided that other conditions were correct.

#### 3.4.3. Shaking/Oxygenation of the Culture

The access to O_2_ was also crucial for achieving adequate pigment concentrations. Molecular oxygen is a direct reactant for BV formation by HO1. In addition, excellent access to this reactive is also necessary to maintain an active aerobic metabolism, providing the reducing power (NADPH) required to couple the two synthesis reactions. Thus, high oxygen transfer to the culture medium, achieved at 250 rpm, resulted in the highest PCB concentration in the flask assays, with a value near 2 mg/L. On the other hand, oxygen deficit in the culture almost completely aborted pigment synthesis (Figure 5c).

Based on all the above data, the optimal pigment synthesis conditions were temperature at 28 °C, initial OD_600_ of 0.5, IPTG concentration of 0.1 mM, and agitation at 250 rpm.

#### 3.4.4. OD_600_ at the Induction Point

After verifying the relevance of the proximity of the stationary phase and metabolic burden for the coupling of the biosynthetic pathway, the effect of the OD_600_ at the induction point was analyzed. The results reported in this section are consistent with previous findings, indicating that the proximity to the stationary phase inhibits pigment synthesis and triggers its degradation. When the cultures were induced at an OD_600_ above 0.8, despite showing a rapid onset of PCB synthesis, it was maintained for only 3–4 h. On the other hand, a low initial OD_600_ (below 0.2) resulted in a delay of 2–3 h before the color appeared in the pellet, likely due to the difficulty of accumulating pigment during the early exponential phase when the growth rate is higher than the pigment synthesis rate (Figure 5d). After the initial appearance of PCB, synthesis remained constant without degradation, but with the drawback of deficient cell concentration compared to higher OD_600_ assays. Finally, it results in an inadequate PCB titer. Therefore, induction OD_600_ of 0.5 was ideal for balancing cell concentration, metabolic burden, and detachment from the stationary phase, resulting in maximum pigment concentration in the culture (Figure 5d,e).

### 3.5. PCB Biosynthesis in Batch Bioreactor

**Figure 6:**
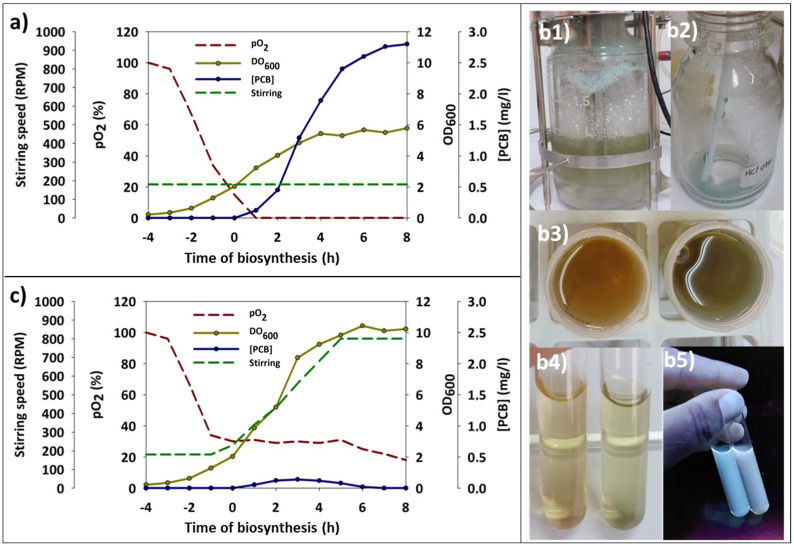
Time-course plots of the main parameters monitored in the bioreactor batch assays. (**a**) Details from the variable stirring assay. Blue foams formed inside the bioreactor (1) and recovered from the gas exhaust (2). Appearance of the supernatant retrieved from the control culture (left) and the assay (right) after 8 h of synthesis (3,4). Red fluorescence of the synthesized PCB (phycocyanobilin) under UVA light (354 nm). (**b**) Parameter evolution at constant stirring and variable pO_2_. (**c**) Parameter evolution at constant pO_2_ and variable stirring. Negative values on the X-axis represent hours before the induction point.

Once the experiments were optimized on a small scale, PCB production was scaled up and assayed in a controlled batch bioreactor. In this new format, the effect of some system-specific variables was investigated. In these systems, the availability of oxygen (pO_2_) in the culture depends on the air inlet flow rate and the stirring speed of the paddle system. The proper setting of these variables was crucial for replicating and scaling the shake flask results. The MM9 medium was used for this set of assays as it offered the highest synthesis levels for both BV and PCB during optimization in shake flask assays. The pH remained 6.8 to 7.2 throughout the experiment due to the phosphate buffer. The induction temperature was 30 °C, decreasing to 28 °C after the first 4 h since the induction. The induction OD_600_ was 1.5. A first assay was conducted to replicate the results obtained in the shake flask. In this case, the air inlet flow rate was kept at 3 L/min, and agitation was kept constant at 200 rpm during the assay. Under these conditions, the pO_2_ dropped to 0 even before induction was triggered. However, good synthesis levels above 2.5 mg/L of PCB were obtained, and a final DO_600_ of 6 units was reached (Figure 6a). A second approach was carried out, aiming to maximize the amount of available oxygen. In this case, the same experiment was repeated by supplying air at 3 L/min while maintaining the pO_2_ at a stable value of 30% by progressively increasing the stirring speed (PID cascade). Surprisingly, PCB levels in the biomass dramatically declined in these conditions, and the cell pellets remained practically gray throughout the experiment.

Nevertheless, as the induction time progressed, the culture medium had a progressive pigment accumulation, even forming blue foams collected through the exhaust gas funnel (Figure 6(b1,b2)). On the other hand, the final OD_600_ was almost double compared to the previous assay due to high oxygen availability (Figure 6c). Analysis of cell-free growth media recovered 8 h after the induction point exhibited a deep blue-green color compared to a control sample obtained from a shake flask without induction (Figure 6(b3,b4)). This blue-green sample was exposed to UVA light and showed a red-shifted fluorescence compared to the control medium, which maintained a pure white basal fluorescence (Figure 6(b5)). These findings suggest that *E. coli* BL21 (DE3) was actively or passively excreting PCB into the media.

**Figure 7:**
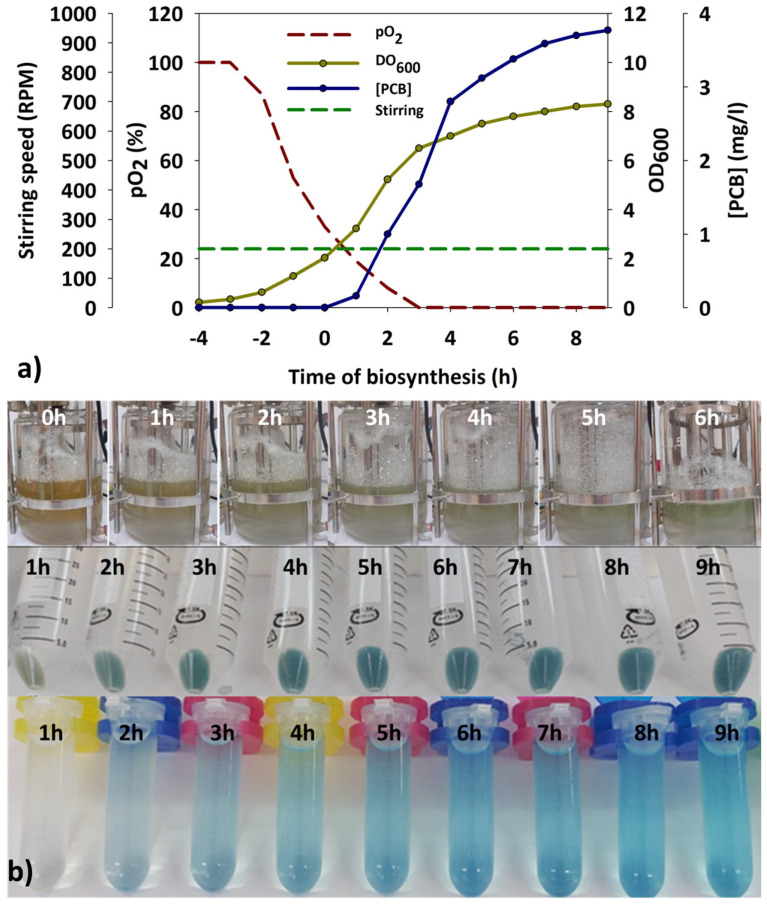
(**a**) Time-course plot of the main parameters monitored in the assay in which maximal PCB (phycocyanobilin) titers were achieved. Negative values on the X-axis represent hours before the induction point. (**b**) Appearance of the batch bioreactor (above), harvested pellets (center), and acidified methanolic extracts (below) at various time points after induction.

From these results, it was suggested that mechanical stress derived from the increasing stirring might alter the permeability of the *E. coli* BL21 (DE3) membranes and allow the synthesized pigment to be released. In addition, this stress could lyse gradually some cells, increasing the pigment release. To verify this phenomenon, the oxygen flow rate was increased from 3 to 6 L/min, while a gently stirring speed of 200 rpm was again maintained. In this way, an increase in available O_2_ was expected while avoiding the detrimental effects of the high stirring speed. Under these conditions, a higher pigment concentration in the biomass was achieved, reaching a value of 3.8 mg/L (Figure 7a,b). Indeed, the PCB release into the medium was virtually avoided following this strategy.

## 4. Discussion

In recent years, there has been a surge in the heterologous synthesis of bilins and PBs. Several studies have successfully synthesized both BV [20,21] and PEB [22,23], and PCB [10,11,12,13,14,15,16] in *E. coli* BL21 (DE3). Regarding the yield, the results reported in these works are highly variable, with production levels ranging from 3.0 to 184.2 mg/L [10,16]. In some of these studies, the genome of the BL21 (DE3) strain has even been modified to enhance the metabolic pathways for heme production as the primary precursor of these pigments and repress the genes involved in some of its degradation pathways [12,13,16]. In other studies, the wild-type *E. coli* Nissle 1917 strain has been used positively as an expression host following genetic manipulation. This strain can incorporate extracellular heme through a specific receptor/transporter (ChuA), thereby increasing the availability of the precursor [21,24]. In addition to the yield variability, these works show surprisingly variable results regarding the optimal parameters described for the production process. The present study offers valuable insights into the dependencies among key parameters influencing PCB synthesis rate and their impact on the host’s metabolism. On one hand, the metabolic state of the host seems crucial for achieving sustained pigment synthesis. In this sense, temperatures between 25 and 30 °C, combined with proper access to O_2_ and abundant and assimilable carbon sources such as glycerol, resulted in an extended exponential phase and a considerable improvement in the rate synthesis and the stability of the pigment. Under these conditions, the host can maintain a high percentage of its electron carriers in a reduced state (NADPH), which are directly used to reduce exhausted FNR and continue supplying electrons to HO1 and PCYA. Simultaneously, this reducing power can keep the heme synthesis pathway active, where some enzymatic steps are also NADPH-dependent [25].

In contrast, the approach to the stationary phase or any other source of stress halts the synthesis and, if prolonged, triggers a fast degradation process of the freshly synthesized pigment. In the obtained results, synthesis times longer than 8–10 h at 28 °C progressively returned the cells to the control phenotype, regaining their initial whitish appearance. BV and PCYA are exogenous molecules to the metabolism of *E. coli* BL21 (DE3), so the metabolic pathways used by the host to degrade them and the reason for their activation when approaching the stationary phase are still unknown [26]. On the other hand, the effect of the inducer on PCB biosynthesis has been highlighted. While IPTG concentrations below 0.1 mM resulted in excessively slow synthesis of HO1 and PCYA, concentrations above 0.3 mM gravely increased the host’s metabolic burden. This was likely due to the overwhelming focus of the BL21 (DE3) strain on the mass production of recombinant proteins, which ultimately damaged the cells by precipitating as inclusion bodies.

In summary, the availability of O_2_, temperature, and the concentration of the inducer definitively influence the intensity of pigment production and the temporal duration of the biosynthetic process until the maximum concentration is reached. Thirdly, adding possible biosynthetic precursors for BV and PCB synthesis produced many results. Similar to other works, the presence of ALA as a heme precursor only generated slight differences in PCB production compared to the reference culture [10]. Other potentially valuable precursors such as NADP^+^ or VC did not generate differences, contrary to what is suggested in other studies in which both ALA and VC did improve the synthesis capacity, but not nicotinic acid [11]. The addition of 10 μM of HEM caused a marked inhibition of bacterial growth and PCB synthesis, indicating a certain degree of toxicity and metabolic burden for the host. This result diverges from that obtained with *E. coli* Nissle 1917, where the host could assimilate and incorporate extracellular heme as a precursor [24]. Based on our results, among all the precursors tested, only GA, and to a lesser degree ALA, yielded significant improvements, not in the synthesis rate itself, but in maintaining it and preventing the degradation of the freshly synthesized pigment [27].

Further research will determine why adding this amino acid generates a notably positive effect while ALA, a subsequent metabolite in the heme synthesis pathway, partially loses this effect. Lastly, it is necessary to highlight the potential connection detected in our results between the increased stirring speed (mediated by paddles) and the release of the fresh pigment into the culture medium. Several studies have previously reported the accumulation of both BV and PEB in the culture medium, however none of them offer an explanation for the cause of this release [20,23]. This is the first report that suggests a possible dependency between both parameters. The results reflect that O_2_ availability significantly improves the synthesis rate of PCB. However, when O_2_ was supplied by increasing the paddle speed, it caused mechanical stress, and the cells tended to excrete the pigment into the medium, probably due to membrane weakening and the subsequent cell death over the induction hours. 

In summary, this study describes optimizing the heterologous synthesis of PCB in *E. coli* BL21 (DE3), achieving a maximum value of 3.8 mg/L. This value refers only to the pigment in the host biomass. However, it has been confirmed that the pigment location can be altered under specific situations (e.g., high paddle stirring), resulting in a significant concentration of excreted PCB into the medium or form foams. Consequently, this study contributes to the current understanding of the molecular mechanisms that control the synthesis, degradation, and heterologous mobilization of PBs in this host. These tests will facilitate the industrial production of these exciting molecules, making their application and commercialization possible soon.

## 5. Conclusions and Future Perspectives

In the reported work, we successfully coupled the enzymatic pathway and optimized the parameters for PCB synthesis in the standard host, *E. coli* BL21 (DE3). The best production results were achieved using a modified medium rich in glycerol and glutamic acid (MM9) in a 2 L batch bioreactor, oxygenated at 6 L/min with constant agitation at 200 rpm. The process was maintained at temperatures between 28 and 30 °C to maximize synthesis, and induction was performed by adding 0.1 mM IPTG when the OD_600_ reached 1.5 units. Under these conditions, a maximum PCB concentration of 3.8 mg/L was obtained 8 h post-induction. Although this production value is modest compared to yields reported in other studies, the parallel results of this study open new avenues for improving process efficiency. Despite suggestions from other studies that improving precursor availability is the key to increasing process yield, our work has demonstrated a strong dependence—equal to or more definitive than precursor availability—on dissolved O_2_ and the host’s ability to sustain PCB synthesis over time and avoid degradative processes associated with entry into the stationary phase.

On the other hand, this bioreactor model can maintain the dissolved O_2_ at a set point by raising the impeller speed while maintaining a constant airflow rate. However, while the amount of available O_2_ is maintained, this rise in mechanical stress leads to a disruption in PCB accumulation, causing host cells to excrete, either voluntarily or involuntarily, the pigment rather than store it. Further research is required to enhance O_2_ availability for the host without inducing mechanical stress. Designing an alternative sparging system for the bioreactor would enable the isolation of both variables (rpm and pO_2_) and assess how much the process yield can be improved by minimizing oxygen limitation. Such research is essential for the future large-scale industrial production of these pigments in a heterologous system.

## Figures and Tables

**Table 1:** Reagents and concentrations used in the three growth media assayed.

	LB (g/L)	TB (g/L)	MM9 (g/L)
**Tryptone**	10.0	12.0	
**Yeast extract**	5.0	24.0	
**Casein peptone**			10.0
**NaCl**	10.0		0.5
**Glutamic acid**			2.5
**Glycerol**		5.0	30.0
**Na_2_HPO_4_·12H_2_O**			8.9
**KH_2_PO_4_**			4.0
**CaCl_2_·2H_2_O** (after autoclaving)			0.04
**MgSO_4_·7H_2_O** (after autoclaving)			0.37
M9 trace metals 1000X * (after autoclaving)			1X

* M9 trace metals composition is described in Table S1.

## Data Availability

The authors will make the raw data supporting this article’s conclusions available upon request.

## Supplementary Materials

The following supporting information can be downloaded at https://www.mdpi.com/article/10.3390/genes15081058/s1. Figure S1: Sequences after codon optimization for the four genes used in this work. Table S1: Composition of metal stock 1000X of MM9 medium. Figure S2: HPLC chromatograms of PCB and BV. Standards and samples extracted from *Escherichia coli* BL21 (DE3) are overlapping. Figure S3: Time-course analysis via SDS-PAGE (20%) of soluble (Fs) and insoluble (Fi) protein fractions from *E. coli* BL21 transformed with pET-*pcyA*^2^/*ho1*^2^.
