# Supplementary material for: Optimization of Phycocyanobilin Synthesis in E. coli BL21: Biotechnological Insights and Challenges for Scalable Production
Source: Genes (Basel). 2024 Aug 12;15(8):1058. doi: 10.3390/genes15081058 (PMC11353606; doi:10.3390/genes15081058)
Supplement: Supplementary file 1 [file genes-15-01058-s001.zip › genes-3142756-supplementary.pdf]

Figure S1. Sequences after codon optimization for the four genes used in this work

ho1[1]

ATGGGAGCTAATTTAGCAACAAAACTAAGGGAGGGCACCAAAAAGGCCCACACCATGGCGGAGAACGTCGGTTTTGTG
AAATGCTTTTTGAAGGGTGTTGTTGAAAAAAACTCCTACCGCAAGCTGGTTGCGAACCTGTACTTCGTGTATAGCGCA
ATGGAAGAGGAGTTGGAACGTCATCGCGATAATGACAAAATCGCTGGTATTTACTTTCCAGTTCTGAATCGCAAGACG
AGCCTGGAGCGCGACTTGGCCTTCTACTATGGTGAGGACTGGCGTCAACAAATTCAGCCGTCGAAAGCGGCGCAAAGC
TACGTGGCGCGTATCAGAGAAGTGAGCAATACCGCTCCGGAATTGCTGGTGGGTCACGCATATACCCGTTATCTGGGT
GATCTGAGCGGTGGTCAGATCCTGAAGACGATTGCCCAACGTGGCATGAACCTGGACGGCGCAGGCACTGCGTTCTAC
GAATTTGAAGCAATTGAGGACGAGAAGGCTTTCAAACAGACCTATCGTCAGGCAATGGATACCCTGCCGGTTGATGAG
GCCACGGCTGATCGTATCGTCGAGGAAGCGAACGACGCGTTCGGCATGAATATGGCCATGTTCCAGGAGTTAGAAGGC
AACCTCGTGGCGGCGATCGGCAAAATGTTTTTCAACTCTCTGACCCGCCGTTTTGTTCGTGGTTCCACCGAATTGGCG
ACCGCGGCGGAATAA

pcyA[1]

ATGTCCTCCAGCACGCAAGTCGGCCTCAAAGAACAGCTGCATCCCTTGATTCGGGATTTGGCCACAGGCATCGAAGCG
ACTTGGCAGCGGTGGCTGAACCTCGAGCCCTACGCGGCGATGCCAGCAGATTTGGGTTACATCGAAGGCAAGCTAGAA
GGCGAGCGGCTCCAGATTGAAAATCGCTGCTACCAAAGCCGGGAGTTTCGCAAACTGCACCTCGAGCTAGCACGGGTT
GGCAACAACCTCGATATCCTCCACTGTGTCCTGTTTCCCCGAACAACTTTTGATCTGCCGATGTTTGGCGCAGACTTG
GTCGGCGGCCGGGGTCAGATCAGTGCGGCGATTGTCGATCTCTCACCGACTACGATTGCCCGAGAACTTTCCAACGAT
TACATCGCAGGTTTGACGGCGTTGCCCAATCCAACCTTTCAAGGCCTGCGGGAGTTACCCACGTGGGGTGATATTTTC
TCGTCCTTCTGTCTGTTTATTCGCCCCGGGTCACCCGAGGAAGAAGCGGCCTTCCTCGATCGCGCCTTGGGCTTTTTG
CAAGTCCATTGTCAACAGGCAGCGGCAGCTACAGCGCTGACCGACCCAGAGGCGATCGCCACCGTGCTCGAGCAGCAG
CGCTACTACTGCGAGCAGCAGCGACGCAATGACAAAACCCGTCGCGTCTTGGAAAAAGCCTTTGGTGATGACTGGGCC
GATCGCTATATGACGACCATGCTCTTTGATTTGCCCAGTGATTGA

ho1[2]

ATGAGCGTTAATCTGGCCAGTCAGCTGCGTGAAGGTACCAAAAAATCACATAGCATGGCAGAAAATGTGGGCTTTGTT
AAATGCTTTCTGAAAGGCGTGGTGGAAAAGAATAGCTATCGTAAACTGGTGGGCAATCTGTATTTTGTGTATAGTGCA
ATGGAAGAAGAAATGGCAAAATTCAAAGATCATCCGATTCTGAGCCATATCTATTTTCCGGAACTGAATCGTAAACAG
AGTCTGGAACAGGATCTGCAGTTTTATTATGGCAGTAATTGGCGTCAGGAAGTTAAAATTAGTGCCGCCGGTCAGGCC
TATGTGGATCGCGTGCGCCAGGTTGCAGCCACCGCACCTGAACTGCTGGTTGCACATAGCTATACCCGCTATCTGGGC
GATCTGAGTGGTGGTCAGATTCTGAAAAAGATTGCACAGAATGCAATGAATCTGCATGATGGTGGTACCGCCTTTTAT
GAATTTGCCGATATTGATGATGAGAAAGCATTCAAAAACACCTATCGTCAGGCCATGAATGATCTGCCGATTGATCAG
GCAACCGCAGAACGCATTGTGGATGAAGCCAATGATGCATTTGCAATGAATATGAAAATGTTCAACGAGCTGGAAGGT
AATCTGATTAAGGCCATTGGCATTATGGTGTTTAATAGTCTGACCCGCCGTCGTAGTCAGGGTAGCACCGAAGTGGGT
CTGGCCACCAGCGAAGGCTAA

pcyA[2]

ATGGCAGTGACCGATCTGAGCCTGACCAATAGCAGTCTGATGCCGACCCTGAATCCGATGATTCAGCAGCTGGCACTG
GCAATTGCCGCCAGCTGGCAGAGTCTGCCGCTGAAACCGTATCAGCTGCCGGAAGATCTGGGCTATGTGGAAGGTCGC
CTGGAAGGTGAAAAACTGGTTATTGAAAATCGCTGCTATCAGACCCCGCAGTTTCGCAAAATGCATCTGGAACTGGCA
AAAGTTGGCAAAGGCCTGGATATTCTGCATTGTGTGATGTTTCCGGAACCGCTGTATGGCCTGCCGCTGTTTGGCTGT
GATATTGTGGCCGGCCCGGGCGGCGTTAGTGCAGCAATTGCCGATCTGAGCCCGACCCAGAGTGATCGCCAGCTGCCG
GCAGCATATCAGAAAAGTCTGGCCGAACTGGGCCAGCCGGAATTTGAACAGCAGCGCGAACTGCCGCCGTGGGGTGAA
ATTTTTAGCGAATATTGTCTGTTTATCCGTCCGAGCAATGTGACCGAAGAAGAACGCTTTGTTCAGCGTGTTGTGGAT
TTTCTGCAGATTCATTGTCATCAGAGTATTGTTGCAGAACCGCTGAGTGAAGCCCAGACCCTGGAACATCGCCAGGGC
CAGATTCATTATTGTCAGCAGCAGCAGAAAAATGATAAAACCCGCCGCGTTCTGGAAAAAGCATTTGGTGAAGCCTGG
GCCGAACGCTATATGAGTCAGGTGCTGTTTGATGTTATTCAGTAA

Table S1. Composition of metal stock 1000X of MM9 medium

Reactive	1000X Stock concentration (mM)
$FeCl_3 \cdot 4H_2O$	31.00
$MnCl_2 \cdot 2H_2O$	6.20
$ZnCl_2$	0.80
$CoCl_2 \cdot 6H_2O$	0.76
$CuCl_2 \cdot 2H_2O$	0.46
$NH_4Mo_7O_{24} \cdot 4H_2O$	0.30

Figure S2. HPLC chromatograms of PCB and BV. Standards and samples extracted from *E. coli* BL21 (DE3) are overlapping

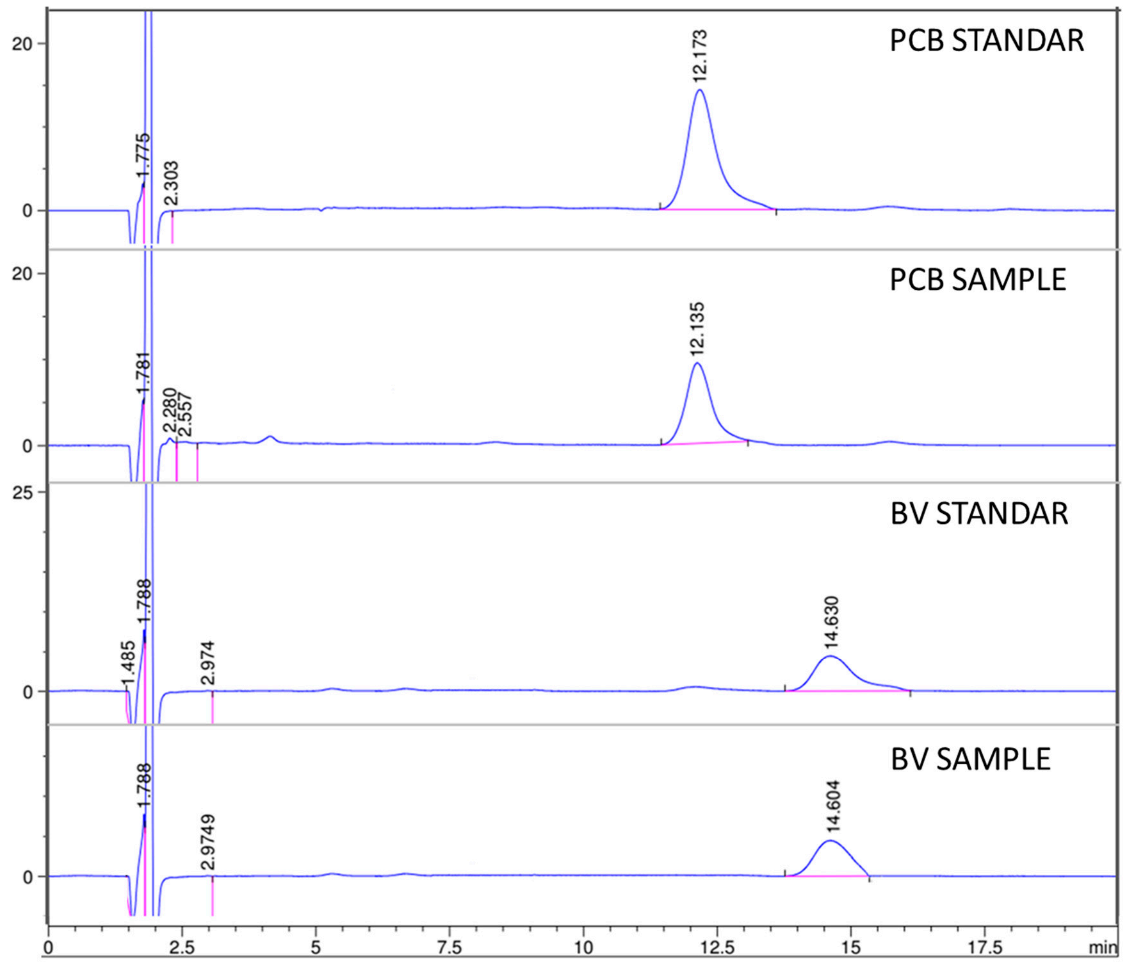

Figure S3. Time-course analysis via SDS-PAGE (20%) of soluble (Fs) and insoluble (Fi) protein fractions from *E. coli* BL21 (DE3) transformed with pET-*pcyA*[2]/*ho1*[2]. At the top, the coloration of the cellular pellet from which each sample was obtained is displayed. Conditions for the biosynthetic phase were 34°C, 180 rpm shaking, 0.3 mM IPTG induction, and an induction OD_{600} of 0.5. Insoluble fractions of recombinant HO1 and PCYA are highlighted with red circles*.

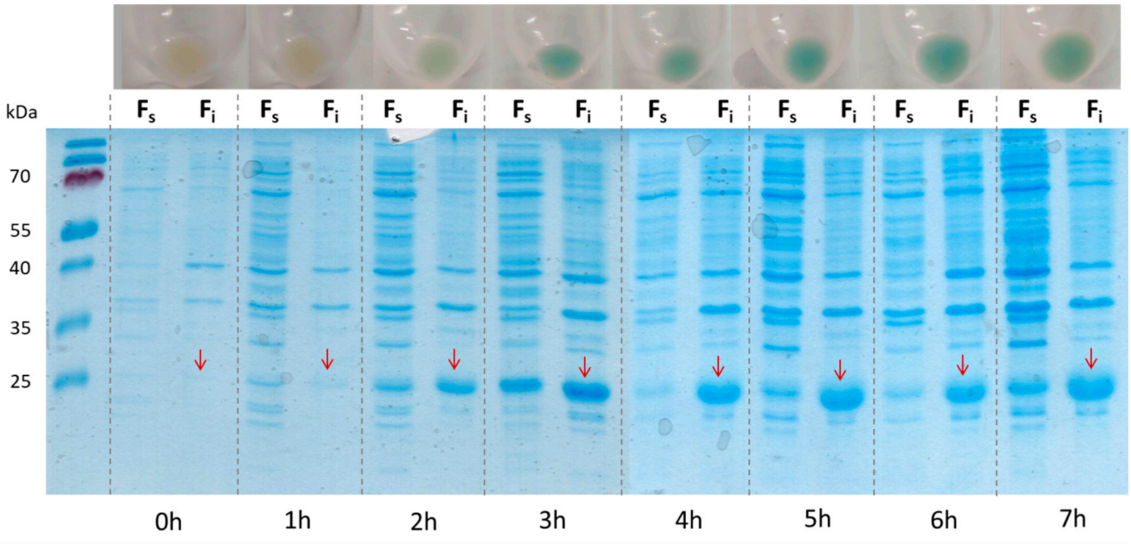

*Note the impossibility in distinguishing via SDS-PAGE the protein bands of HO1 and PCYA due to their similar molecular weights of 27 and 28.1 kDa, respectively
